# Positively Charged Organosilanes Covalently Linked to the Silica Network as Modulating Tools for the Salinity Correction of pH Values Obtained with Colorimetric Sensor Arrays (CSAs)

**DOI:** 10.3390/s24020417

**Published:** 2024-01-10

**Authors:** Andrea Pastore, Denis Badocco, Luca Cappellin, Mauro Tubiana, Paolo Pastore

**Affiliations:** Department of Chemical Sciences, University of Padua, Via Marzolo 1, 35131 Padua, Italy; andrea.pastore.1@phd.unipd.it (A.P.); denis.badocco@unipd.it (D.B.); luca.cappellin@unipd.it (L.C.); mauro.tubiana@unipd.it (M.T.)

**Keywords:** colorimetric sensor arrays, pH measurement, chemical sensors, real-time monitoring, seawater, sol–gel

## Abstract

Seven increasing levels of water salinity from 0.029 to 0.600 M (as NaCl) were used to investigate the dependence of pH measurement, performed using colorimetric sensor arrays (CSAs), on ionic strength. The CSAs were arrays of sensing spots prepared in the form of sol–gel-embedding Bromothymol Blue (BB) and Bromocresol Green (BCG) in a porous nitrocellulose support. The support was impregnated over the entire thickness (≈100 µm), allowing for the signal (Hue) acquisition on the opposite side to the contact with the sample solution. Three CSAs were prepared, M1, M2, and M3. M1 contained a free cationic surfactant, hexadecyltrimethylammonium p-toluenesulfonate (CTApTs), for modulating the ${pK}_{a}$ of the indicators. In M2, the surfactant dimethyloctadecyl[3-(trimethoxysilyl)propyl]ammonium chloride (DTSACl) was covalently bonded to the sol–gel. M3 was prepared like M2 but using a larger amount of ethanol as the solvent for the synthesis. The modulation of the CTApTs or the DTSACl concentration enabled the tuning of the ${pK}_{a}$. In general, the ${pK}_{a}$ modulation ability decreased with the increase in salinity. The presence of a surfactant covalently linked to the backbone partially reduced the competitiveness of the anionic species, improving the results. Nevertheless, the salt effect was still present, and a correction algorithm was required. Between pH 5.00 and 12.00, this correction could be made automatically by using spots taken as references to produce sensors independent of salinity. As the salt effect is virtually absent above 0.160 M, M2 and M3 can be used for future applications in seawater.

## 1. Introduction

pH is a key parameter that can be used as a probe for different phenomena like food spoilage [1,2,3,4,5], fermentation [6,7,8,9], ocean acidification [10,11,12,13], industrial processes, and so on [14,15,16,17,18]. Potentiometric determination with a glass electrode is traditionally the most important method used for pH measurement [19]. Nevertheless, it is characterized by various limits, such as the signal dependence on the ionic strength due to the variation of the liquid junction potential [20], the alkaline and the acidic errors [21,22], and the slow response time in low-conductive solutions and low temperatures [19,20]. The determination of the pH value in highly saline solutions, such as seawater, is still a critical issue [23], as the buffer solutions usually used for the calibration of the glass electrode are characterized by an ionic strength very different from that of the seawater samples [19]. To avoid large errors in the discrimination of pH, specific buffers must be prepared, but they are not stable [24]. In the case of mineral water, especially for conductivity values lower than 200 µS/cm, the correction is quite difficult [25]. Moreover, contamination of the sample is possible through the liquid junction with the sample, owing to the diffusion of the KCl present in a large concentration in the reference electrode [20]. On the other hand, the need to perform in-field measurements contrasts with the batch nature of the glass electrode [13]. In the last few years, several efforts have been made to improve pH measurement [3,26,27]. Colorimetric sensor arrays (CSAs) [14,28,29,30] represent an economic and simple way to achieve this purpose [14,31]. Several colorimetric devices have been proposed. From the simple litmus paper [32,33], in the last 15 years, a great evolution of more and more complex polymer matrices has widened the audience and the attention of the scientific community concerning CSAs [26,34,35]. Nevertheless, they suffer from various issues: errors due to the acquisition of the signal (the solution is interposed between the CCD camera and the CSA), leaching effects or irreversibility [36], slow time response [37], and low accuracy [38]. The first issue is quite difficult to solve [39,40]. Gas bubbles and impurities can determine a large error in the color detection of the CSA. In 2022, the patent “Colorimetric Sensor Arrays (CSAs)”, developed in our research group, was successfully granted [41]. The colorimetric sensor is based on a polymer, working as support (polyvinylidene fluoride, PVDF), a gel matrix deposited on the support, and one or two embedded acid–base indicators [42]. A further necessary additive is a suitably charged surfactant [43]. By tailoring its concentration, it is possible to tune the ${pK}_{a}$ of the indicator to obtain high and homogeneous precision in the entire working interval (0–14). The analytical signal is based on *H* (Hue) from the HSV color space [42,44,45]. The accuracy is dependent on the saline concentration of the test solution [46]. In 1986, Salvatore et al. accounted for the behavior of the dissociation constants of various acid–base indicators in solution [47]. Nevertheless, in complex polymer matrices including surfactants and silanes, the salt effect on the pKa of common acid–base indicators has never been rationalized. In 2022, Pastore et al. proposed a model including five reactions, involving all the components of the CSA [48]. The model was also successfully applied in hydro-alcholic solutions, showing a dramatic change in the working constants. In particular, the thermodynamic constant describing the equilibrium between the indicator and the surfactant, K_S_, is crucial to determine the dependence of the signal on the ionic strength and, thus, the accuracy [48]. The logarithm of K_S_ changed from 6.0 in solution to 4.9 in one of the tested CSAs. These last achievements were the starting points of the rationalization of the salt effect in modified CSAs, prepared in our lab in 2023, including nitrocellulose support and a different precursor used during the sol–gel synthesis. 

In this paper, the influence of the ionic strength was studied concerning its effect on the pH measurement made with a CSA. The sensing spots were supported by a suitable nitrocellulose membrane that can be impregnated over the entire thickness (≈100 µm), allowing for the color reading on the opposite side to the contact with the sample solution. Indeed, the pH reading changed with salinity compromising the accuracy of the results. The use of surfactants covalently bonded to the silica network of the sensing spot (in place of the previous free ones) helped reduce the salt effect. The surfactant used is the dimethyloctadecyl[(3-(trimethoxysilyl)propyl]ammonium chloride (DTSACl), and the sensing spots were prepared also with different amounts of solvents during the synthesis. Anyway, a correction algorithm was required when the ionic strength was lower than 0.16 M. At values larger than 0.16 M, the sensor response was independent of salinity, so its use for applications in which high salinity is present, including seawater, is suggested. A model for the behavior of the ${pK}_{a}$ vs. the concentration of surfactant $C_{S}$ was proposed to obtain an algorithm for the correction of salinity of the pH value. 

## 2. Materials and Methods

### 2.1. Reagents and Instrumentation

Bromocresol green (BCG), Bromothymol Blue (BB), TEOS (≥99%), hexadecyltrimethylammonium p-toluenesulfonate (CTApTs), dimethyloctadecyl[3-(trimethoxysilyl)propyl]ammonium chloride (DTSACl in methanol solution 42%), NaOH, boric acid, phosphoric acid, and acetic acid were provided by Sigma Aldrich. Nitrocellulose (porosity 0.45 µm) was provided by ThermoFisher Scientific (China). The ethanol was from Carlo Erba.

All regressions were performed through MATLAB 2018a by using the iterative “Levenberg Marquardt” algorithm [49]. The pH measurements were carried out at 26 °C with a Hanna Instruments HI11310 glass electrode (Woonsocket, RI, USA), which was calibrated with two Hanna Instruments standard solutions at pH 7.00 and 4.01, respectively. The color acquisition was carried out after immersing the CSAs for 120 s. The color was sampled in a homogeneous central portion of the spot (800–900 pixels). Appropriate programs written with MATLAB were used to extrapolate the RGB coordinates from which it was possible to calculate the HSV values. 

### 2.2. Measurement Cell

**Figure 1:**
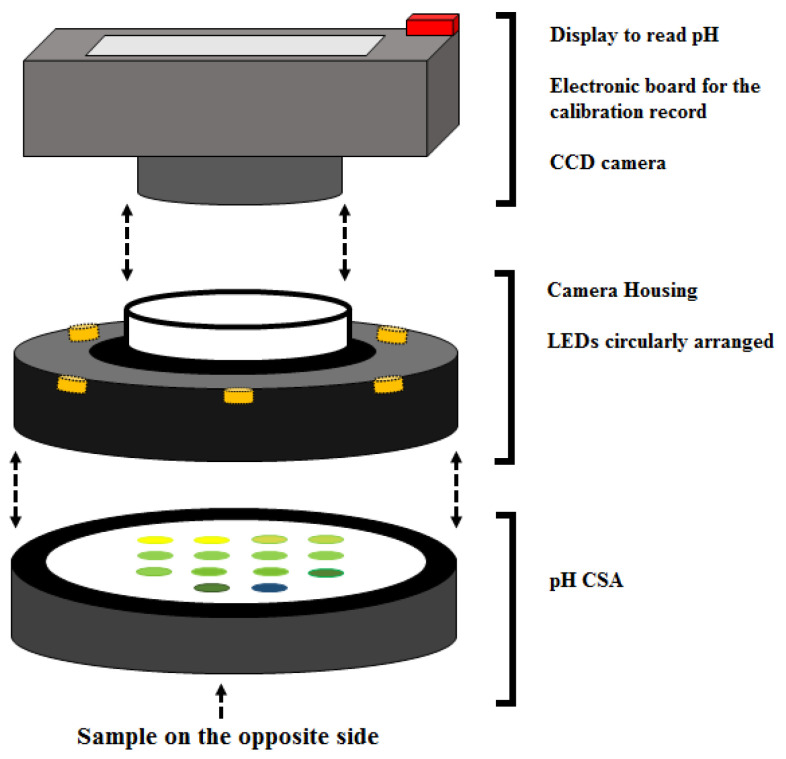
Measurement cell.

The device to detect pH is based on the reading of the *H* parameter from the HSV color space as the analytical signal. The scheme of the measurement cell is reported in Figure 1. The pH meter consists of: (I) a nitrocellulose membrane impregnated with a pH-sensitive gel (77 spots); (II) a CCD camera for the detection of the color; (III) an electronic board containing the calibration profiles in terms of *H* as a function of pH, and (IV) a display to read the pH of the samples.

The shape of the *H* vs. pH profiles is sigmoidal, and it was fitted with the Boltzmann model (Origin 9.0). A cut-off algorithm was used to define the window of the *H* signal in which the spots can be considered active (dotted lines). Since the excursion of each spot was between *H* = 0.120 and 0.605, the first 7% (*H* = 0.120–0.154) and the last 7% (*H* = 0.571–0.605) of the excursion were excluded. ∆pH is the pH interval in which the calibration profile is characterized by an almost constant precision. ${∆pH}_{MAX}$ is a working interval larger than ∆pH that takes into account 86% of the total excursion of the *H* coordinate from the acidic to the basic conditions. The same holds for all the sigmoids (spots). ${pH}_{i}$ is the pH of the inflection point. As demonstrated in another paper [48], ${pH}_{i}$ = ${pK}_{a}$ + const, so for the rest of the discussion, this parameter (easily obtained by the *H* reading) will be used instead of ${pK}_{a}$ to comment on the shift of the calibration profiles as a function of the surfactant concentration and/or buffer concentration. Nevertheless, during the pH measurements, the entire sigmoidal profiles will be used (they are recorded on the electronic board). 

### 2.3. Preparation of Nitrocellulose-Supported Colorimetric Sensors

The CSA consisted of 77 spots (Ø ≈ 3 mm each). The spots with $C_{S}$ = 0, 0.51 M were repeated three times to estimate the repeatability. BCG and BB were characterized by a transition between complementary colors from the HIn (yellow) to the ${In}^{-}$ (blue) forms and were prepared in ethanolic solutions by mixing 203.6 mg and 182.0 mg to 13.55 and 13.51 g of EtOH, respectively. Each spot had a proper value of $C_{S}$, which characterized a specific working interval. The edges of the membrane were glued to an optical plastic window with a commercial adhesive. The color reading was completed on the opposite side to the sample solution, since the gel permeated over the entire thickness of the nitrocellulose membrane and the sample permeated completely, too.

(M1) Spots #1–22: the first row contained BCG at $C_{S}$: 0.511 (three times), 0.345, 0.234, 0.174, 0.118, 0.063, 0 M (three times); the second row contained BB at $C_{S}$: 0.513 (three times), 0.338, 0.242, 0.174, 0.117, 0.064, 0 M (three times). The nitrocellulose membrane was impregnated with a sol–gel solution prepared by acidic hydrolysis of TEOS (14.56 g of TEOS, 7.28 g of Milli-Q water, 0.21 g of HCl 1 N). After 45 min of magnetic stirring, the sol became clear, and the total amount was separated into two vials with 10.08 g of the sol. In one of these, 2.016 g of CTApTs was added. The intermediate concentrations were obtained by a suitable mixture of the two gels. For each $C_{S}$ value, a mixture of 1.04 g of sol and 0.87 g of ethanolic solution of the indicator was prepared. The mixture amount used for each spot was 95 µg. 

(M2) Spots #23–55: the first row BCG at $C_{S}$: 0.508 (three times), 0.331, 0.228, 0.168, 0.121, 0.059, 0 (three times); the second row BB at $C_{S}$: 0.508 (three times), 0.335, 0.224, 0.168, 0.116, 0.071, 0 (three times); the third row: the same as the first. The spots were obtained by a mixture of two sols. Sol_1_ was prepared by mixing under magnetic stirring (45 min) 2.43 g of TEOS, 1.23 of Milli-Q water, 0.03 g of HCl 1 N, and 1.96 g of DTSACl. For each indicator, 2.31 g of Sol_1_ was added to 1.06 of its ethanolic solution. The Sol_2_ was prepared by mixing under magnetic stirring (45 min) 5.00 g of TEOS, 2.66 g of Milli-Q water, and 0.07 g of HCl 1 N. For each indicator, 3.10 g of Sol 2 was added to 2.57 of its ethanolic solution. Finally, the two sols were mixed to obtain the vials in Table 1. 

(M3) Spots #56–77: the first row BCG at the same $C_{S}$ values of M2; the second row BB at the same $C_{S}$ values of M2. To each vial of M2 was added 0.30 g of ethanolic solution.

**Figure 2:**
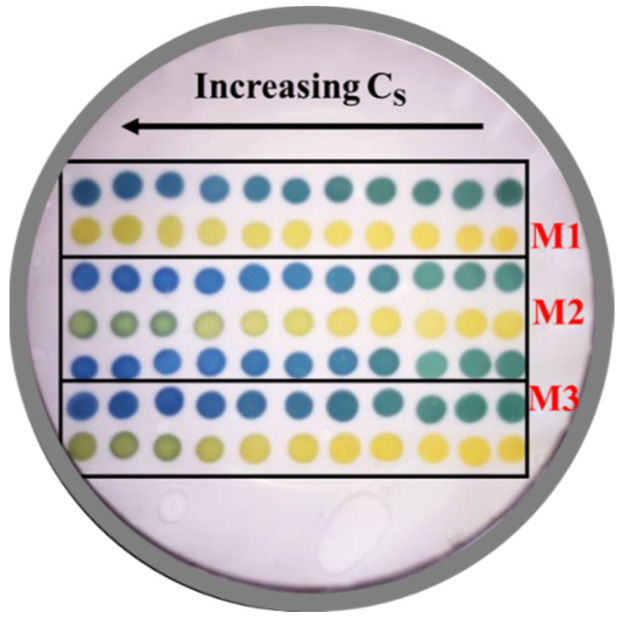
Photo of the CSA at pH = 7.62 (salinity = 0.397 M). BCG: first, third, fifth, and sixth row. BB: second, fourth, and seventh row.

The CSAs were left to age for three days. The CSA was kept under vacuum by using a bag sealer. After the aging period, the CSA was washed with a 0.08 M NaOH solution for 3 h to remove the unreacted precursors and then left at pH = 2 for 2 days. Figure 2 shows a photo of the CSA at pH = 7.62 with a buffer with a saline concentration of 0.397 M. 

### 2.4. Preparation of the Buffer Solutions

Seven salinities (0.029 M, 0.043 M, 0.061 M, 0.109 M, 0.211 M, 0.397, and 0.600 M corresponding to 1.86, 2.75, 3.91, 6.98, 13.52, 25.43, and 38.43 g/L) were tested to quantify the ${pH}_{i}$ shift due to the competitiveness among the various anionic species. The buffers were prepared by using boric acid, phosphoric acid, and acetic acid and adding an increasing amount of NaCl to obtain the required salinity. The starting point was the mixture of boric, acetic, and phosphoric acids with NaCl. The pH of the first buffer was around 2.4. By adding small amounts of NaOH, the pH moved toward more basic pH values. The addition of NaOH was completed by taking into account dilution effects <5%. For more acidic pH values, a small amount of HCl was added. In this case, the total chloride concentration was adjusted by reducing the amount of NaCl in the starting point mixture. The conductivity of the solutions was measured with the conductivity probe (HI763100, Hanna Instruments, Woonsocket, RI, USA). 

### 2.5. Electronic Board

The *H* acquisitions were obtained after immersing the CSAs for 120 s. The color was sampled in a homogeneous portion of the spot (≈800 pixels). Appropriate programs, written with MATLAB, were used to extrapolate the RGB coordinates (median of 800 values for each spot), from which it was possible to calculate the *H* value. All regressions were performed with MATLAB using the iterative “Levenberg–Marquardt” algorithm [49]. Each spot was characterized by its calibration profile, i.e., a different working interval due to the different concentrations of the surfactant. The precision and the slope of the *H* profile depended on the pH [40]. At a given pH value, not all the spots were “active” and their contribution to the pH value was weighted for the slope and the variance regression obtained by the fitting of the calibration profile. The readout was therefore a weighted mean value ($\bar{pH}$) of many acquisitions (${pH}_{i}$) of many spots. The weight ($w_{i}$) was obtained as:

(1) $$w_{i}=\frac{{\left(1/s_{{pH}_{i}}\right)}^{2}}{\sum_{i}^{N}{\left(1/s_{{pH}_{i}}\right)}^{2}}$$

$s_{pHi}$ was approximatively equal to the ratio $\frac{s_{y/x}}{b}$ where the $s_{y/x}$ and *b* parameters were the regression standard deviation and the slope of the calibration profile of the spot, respectively. The mean $\bar{pH}$ reported by the display was:

(2) $$\bar{pH}=\sum_{i}^{N}w_{i}\cdot {pH}_{i}$$

where *N* was the number of spots, and the weighted standard deviation $s_{\bar{pH}}$ is:

(3) $$s_{\bar{pH}}=\sqrt{\frac{\sum_{i}^{N}{w_{i}({pH}_{i}-\bar{pH})}^{2}}{\frac{M-1}{M}\cdot \sum_{1}^{N}w_{i}}}$$

*M* is the number of non-zero weights (active spots). $\sum_{1}^{N}w_{i}$ is equal to 1. The pH reported on the display was the median value obtained from 10 consecutive acquisitions of 1 s (10 values of $\bar{pH}$). This value was also accomplished by the median of 10 values of the weighted standard deviation. The median of the weighted standard deviation was around 0.05 pH units.

## 3. Results and Discussion

**Figure 3:**
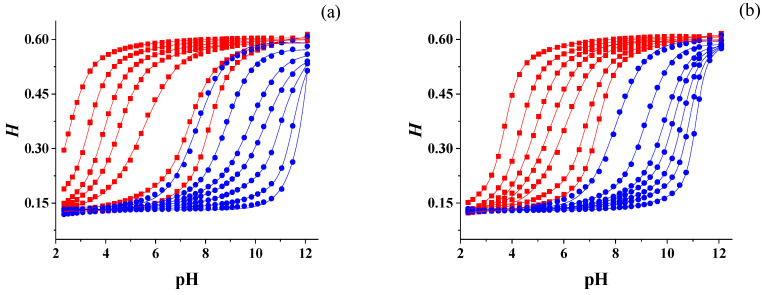
H vs. pH profiles for M2 containing BB (●) and BCG (■). The pH buffers were prepared at 0.061 M (**a**) and 0.397 M (**b**) NaCl.

Figure 3 shows the calibration profiles of 14 spots of the M2 sensor array (as an example) in terms of H vs. pH. Seven of them contained BB (●) and seven contained BCG (■). The surfactant concentration in the spots decreased from left to right. The two representations referred to the response of the sensor to pH buffers prepared at different ionic strength values: namely, 0.061 M (a) and 0.397 M (b), respectively. There was a quite evident pH shift of both indicators, but it was more important for BCG. The pH shift indicated a variation of the ${pK}_{a}$ caused by a series of factors connected to the interaction of the anionic species present in the buffer and the deprotonated silanols of the sol–gel with the cationic head of the surfactant confined inside the polymer network. These interactions, in turn, were reflected in the different behavior of the acid–base indicator, that is the variation of the ${pK}_{a}$ [43,48]. The overall conclusion was that if the sensor was calibrated in the conditions of Figure 3a, but the real sample had the ionic strength of Figure 3b, the pH measurement was wrong. For this reason, a correction strategy was necessary.

**Figure 4:**
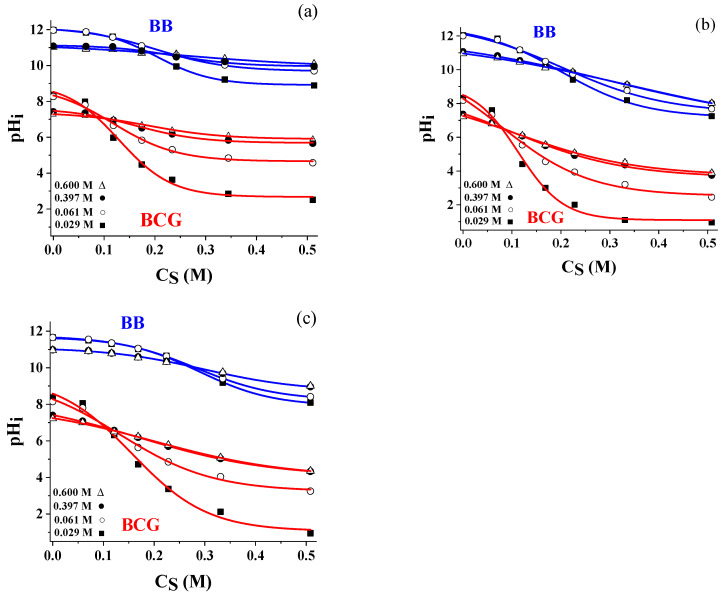
${pH}_{i}$ values vs. $C_{S}$ for M1 (**a**), M2 (**b**), and M3 (**c**) for three levels of salinity 0.029 M (■), 0.061 M (○), 0.391 M (●), and 0.600 M (∆).

The M1 and M3 arrays had a similar behavior. Around the inflection points, the sigmoids could be interpolated with a straight line leading to a simpler model to correct the pH reading of the CSA as a function of salinity. In particular, the pH of the inflection point of the sigmoids, ${pH}_{i}$, was taken as a reference to model the pH shift. Figure 4 shows the experimental ${pH}_{i}$ values vs. $C_{S}$ for M1 (a), M2 (b), and M3 (c) for four levels of salinity 0.029 M (■), 0.061 M (○), 0.397 M, and 0.600 M (●) fitted with the sigmoidal Boltzmann model (continuous lines). The error bars due to the standard deviation of the ${pH}_{i}$ values (spots repeated three times) were too small to be visible in these figures (<0.05 pH units). In this context, it is important to notice that the data were quite robust, as 800 pixels are read for each spot, and the median value produced the final readout. Considering that 40 buffers were prepared to obtain a single sigmoid, each experimental ${pH}_{i}$ value came from 32,000 data points. The blue and red lines were the fitting curves that could be used as models of the pH variation. 

Figure 4 indicates the following:

- The pH shift due to the surfactant was lower at large salinity values. Referring to Figure 4a, the BCG indicator, for instance, moved ${pH}_{i}$ from pH 8.37 to 2.51 (Δ${pH}_{i}$ = 5.86) at salinity of 0.029 M (■) and from pH 7.43 to 5.65 (Δ${pH}_{i}$ = 1.78) from at salinity of 0.397 M (●). Theoretically, to produce a full-range sensor, the best situation was characterized by the largest pH variation due to the surfactant. This would be advisable at all the ion strengths. A constant trend of the ${pH}_{i}$ values vs. $C_{S}$ (see for instance BB at large ion strength) led to having only one or two active spots, rendering the array useless as it read a limited pH interval.
- BB was less affected than BCG.
- The pH shift due to the salinity was positive for spots with larger $C_{S}$ values and negative for the less concentrated ones, leading to intersection points representing the independence of the measured pH from salinity. These points can be taken as references to correct the dependence on salinity using suitable theoretical models. Considering that the working interval was around 2.50 pH units for each spot, the pH interval in which it is possible to perform an autocorrection with suitable software integrated with the pH-CSA device was between pH = 5.00 and 12.00. To achieve an accurate measurement below pH = 5.00, it was necessary to use another pH indicator or a second device like a conductivity probe.

**Figure 5:**
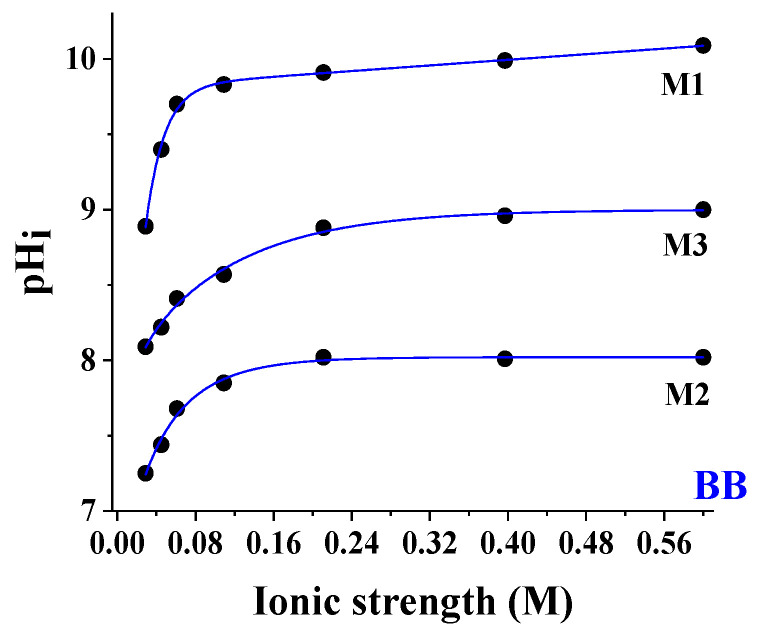
${pH}_{i}$ values (●) vs. ionic strength (M) for M1, M2, and M3 referring to the single spot containing BB having $C_{S}$ = 0.53 M chosen as the worst case (largest pH variation with salinity). T = 25 °C.

**Figure 6:**
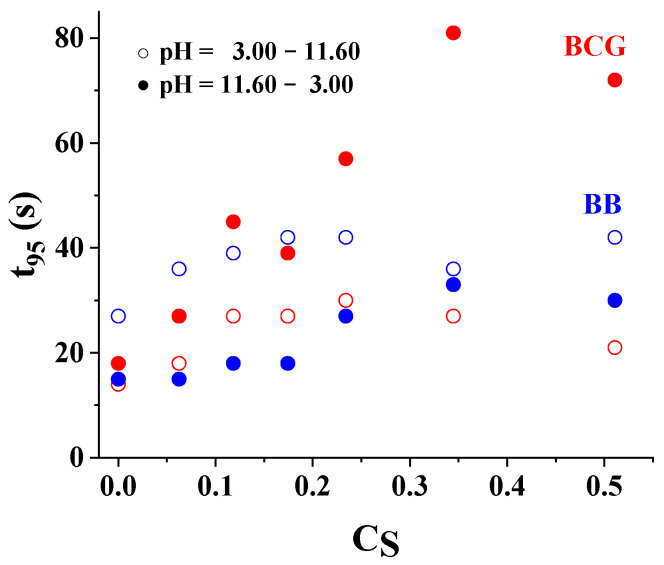
$t_{95}$ values (s) vs. $C_{S}$ for M2. ○ indicates the $t_{95}$ values referred to the transitions between pH = 3.00 and 11.60. The $t_{95}$ values for the reverse pH transition are indicated with ●. Buffer concentration = 0.397 M. T = 25 °C.

Figure 5 describes the shift in the experimental ${pH}_{i}$ values (●) with the buffer concentration for M1, M2, and M3. Here, we referred to the single spot containing BB having $C_{S}$ = 0.53 M chosen as the worst case (largest pH variation with salinity). The salt effect was characterized by a plateau after 0.160 M (Figure 6), so the dependence of the ionic strength for M2 and M3 was negligible and suggested applications in seawater (20–38 g/L) without the need for correction algorithms. Below 0.160 M, the shift due to the salt effect was larger in the case of M1 (1.10 pH units vs. 0.55 pH units in M2). 

The $t_{95}$ values (time required to reach 95% of the acidic/basic H plateau) for BCG and BB spots in M2 vs. $C_{S}$ are reported in Figure 6 at 0.397 M buffer concentration. The open circles (○) indicate the $t_{95}$ values referred to the transitions between pH = 3.00 and 11.60. The reverse transition is indicated with the black circles (●). The behavior in the two senses is not symmetric. Generally, the transition from the acidic to the basic environment was less affected by the surfactant concentration and thus by the pH working interval of the spots. The spots with larger $C_{S}$ values were characterized by slower return times, since the ion pair between the deprotonated form of the indicator and the cationic head of the surfactant represented an obstacle to the diffusion of the protons. 

## 4. Conclusions

Seven increasing levels of water salinity from 0.029 to 0.600 M, prepared with NaCl, were used to investigate the ionic strength dependence of the pH measurement performed by colorimetric sensor arrays (CSAs) based on two acid–base indicators embedded in suitable sol–gel matrices, namely, Bromothymol Blue (BB) and Bromocresol Green (BCG). Three CSA were prepared (M1, M2, and M3) using a porous nitrocellulose sheet as support. The sensing spots impregnated the supporting membrane over the entire thickness (≈100 µm), allowing for the signal (Hue) acquisition from the opposite side of the sample solution. M1 was characterized by the presence of a free cationic surfactant, hexadecyltrimethylammonium p-toluenesulfonate (CTApTs) for the modulation of the ${pK}_{a}$ value of the indicators. This modulation allowed a pH span of 9.47 pH units. In the CSA M2, the surfactant, dimethyloctadecyl[3-(trimethoxysilyl)propyl]ammonium chloride (DTSACl), was covalently bonded to the sol–gel. In this case, the pH span was 11.10 pH units. M3 differed from M2 in the presence of a larger amount of ethanol in the synthesis of the layer. The pH shift due to the surfactant was lower at large salinity values, and this fact limited the pH interval useful for the CSA, although both M2 and M3 showed a sufficient slope. BB was less affected by salinity than BCG, but the pH working interval was lower. The pH shift due to the salinity was positive for spots with larger $C_{S}$ values and negative for the less concentrated ones, leading to intersection points representing situations in which the measured pH is independent of salinity. These points can be taken as references to correct the dependence on salinity using suitable theoretical models. Considering that the working interval was around 2.50 pH units for each spot, the pH interval in which it was possible to perform an autocorrection with suitable software integrated with the pH-CSA device was between pH = 5.00 and 12.00. To achieve an accurate measurement below pH = 5.00, it was necessary to use another pH indicator working at lower pH values. The dependence of M2 and M3 on the ionic strength was negligible after 0.160 M, so the CSA can be used for future applications in the field of seawater in which the salt concentration ranges between 0.400 and 0.600 M. The membrane is stable over months. When it is damaged, it can be easily replaced with another one and screwed into the rest of the compact device. 

## 5. Patents

IT102019000013878, Sensore colorimetrico per misure di pH, Luca Cappellin, Paolo Pastore, Denis Badocco, Andrea Pastore. 

## Figures and Tables

**Table 1:** Composition of the vials used for M2.

BCG	Sol_1_ + EtOH (g)	Sol_2_ + EtOH (g)	BB	Sol_1_ + EtOH (g)	Sol_2_ + EtOH (g)
1	0.939	0	1	0.930	0
2	0.599	0.359	2	0.610	0.352
3	0.405	0.557	3	0.400	0.566
4	0.307	0.653	4	0.296	0.668
5	0.210	0.750	5	0.202	0.763
6	0.103	0.877	6	0.124	0.861
7	0	0.955	7	0	0.947

## Data Availability

Data are contained within the article.
